# Fluid shear triggers microvilli formation via mechanosensitive activation of TRPV6

**DOI:** 10.1038/ncomms9871

**Published:** 2015-11-13

**Authors:** Shigenori Miura, Koji Sato, Midori Kato-Negishi, Tetsuhiko Teshima, Shoji Takeuchi

**Affiliations:** 1 Institute of Industrial Science, The University of Tokyo, 4-6-1 Komaba, Meguro-ku, Tokyo 153-8505, Japan; 2 Takeuchi Biohybrid Innovation Project, Exploratory Research for Advanced Technology (ERATO), Japan Science and Technology (JST), Tokyo 153-8904, Japan

## Abstract

Microvilli are cellular membrane protrusions present on differentiated epithelial cells, which can sense and interact with the surrounding fluid environment. Biochemical and genetic approaches have identified a set of factors involved in microvilli formation; however, the underlying extrinsic regulatory mechanism of microvilli formation remains largely unknown. Here we demonstrate that fluid shear stress (FSS), an external mechanical cue, serves as a trigger for microvilli formation in human placental trophoblastic cells. We further reveal that the transient receptor potential, vanilloid family type-6 (TRPV6) calcium ion channel plays a critical role in flow-induced Ca^2+^ influx and microvilli formation. TRPV6 regulates phosphorylation of Ezrin via a Ca^2+^-dependent phosphorylation of Akt; this molecular event is necessary for microvillar localization of Ezrin in response to FSS. Our findings provide molecular insight into the microvilli-mediated mechanoresponsive cellular functions, such as epithelial absorption, signal perception and mechanotransduction.

## Abstract (web summary)

Microvilli on epithelial cells can sense the surrounding fluid environment, but the regulatory mechanism behind their formation is mostly unknown. Here Miura *et al.* show that fluid shear stress serves as a trigger for microvilli formation via activation of the calcium ion channel TRPV6.

## Graphical abstract

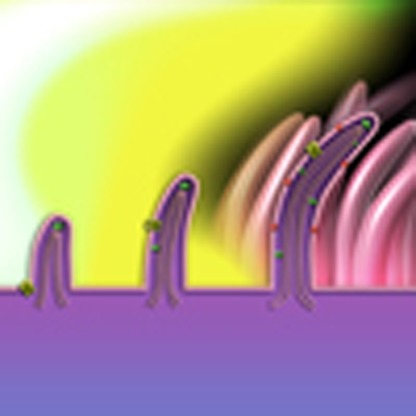

Microvilli are actin-based membrane protrusions that facilitate diverse epithelial functions, including absorption, secretion and mechanotransduction^1^,^2^,^3^. Microvilli also provide greater surface area on the apical side of cells and enable asymmetric localization of membrane transporters, cytoskeletal proteins and enzymes for polarized cellular function^3^. Various cell types, such as cells of the intestinal epithelia, proximal tubule of the kidney, nasal epithelia and placental syncytium^4^, form microvillar surfaces that effectively sense and interact with the fluid environment. In the past decade, intracellular factors, including calcium ions, ezrin/radixin/moesin proteins and ezrin/radixin/moesin-binding protein 50, have been identified as essential factors for microvilli formation^3^,^5^,^6^. Even though the cells make direct contact with fluid flow, previous studies did not focus on the extracellular cues, especially fluid shear stress (FSS), as a trigger for microvilli formation.

Here we uncover that FSS serves as a critical external cue for microvilli formation in placental barrier cells. The placental barrier cells develop thousands of microvilli exposed to the maternal blood in the intervillous space and regulate material transfer between the maternal and fetal blood flow (Fig. 1a). We fabricate a multilayer microfluidic device to analyse material transport through the cells and to observe cellular responses to a broad range of FSS (Fig. 1b,c). Using this device, we show that both BeWo trophoblastic cells and villous trophoblasts form abundant microvilli of varying lengths depending on the flow rate, whereas the cells under static fluid conditions have sparse microvilli. Moreover, we demonstrate that the transient receptor potential, vanilloid family type-6 (TRPV6) calcium ion channel is essential in FSS-induced Ca^2+^ influx and microvilli formation in BeWo trophoblastic cells. We also identify the downstream phosphorylation signalling required for the microvilli formation.

## Results

### Induction of microvilli by FSS in BeWo cells

**Figure 2:**
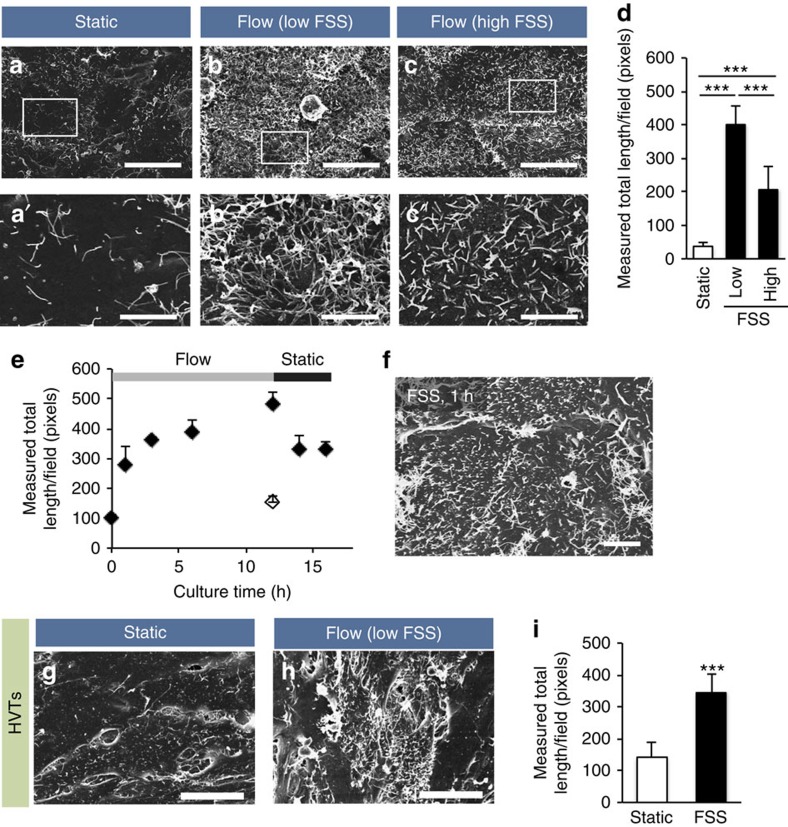
FSS-induced microvilli formation in trophoblastic cells. (**a**–**c**) Scanning electron microscopy surface images of BeWo cells cultured under static or flow conditions. Cells were seeded in the chamber area of the device and cultured overnight with or without medium perfusion (5 μl min^−1^) in both channels. For the flow-exposed cells, images were captured at the centre (low FSS) or inlet (high FSS) area of the chamber. The boxed areas in **a**–**c** are magnified in **a′**–**c′**, respectively. Scale bars, 20 μm (**a**–**c**) or 5 μm (**a′**–**c′**). (**d**) Quantification of microvilli. Total length of microvilli per field was measured from the SEM images (700 μm^2^, five fields) as described in the Methods. The data represent the mean±s.d.; ***P<0.001, analysis of variance. Representative of three independent experiments. (**e**,**f**) Time course analysis of FSS-induced microvilli formation. BeWo cells were cultured under FSS (5 μl min^−1^) for 12 h and then cultured without medium perfusion for additional 4 h. Culture medium was perfused only in the maternal channel/chamber (solid diamonds) or only in the fetal channel (open diamond). The cells were fixed at the indicated time points, and total length of microvilli per field (2,800 μm^2^, five fields) was analysed (**e**). The data represent the mean±s.d. The experiment was repeated twice with similar results. Note that numerous microvillar sprouts were observed 1 h after medium perfusion (**f**). Scale bar, 5 μm. (**g**–**i**) SEM images of HVTs cultured overnight with or without medium perfusion (2 μl min^−1^) in both channels. Representative images (**g**,**h**) were captured at the inlet area of the chamber, and total length of microvilli per field (11,200 μm^2^, five fields) was measured (**i**). Scale bar, 20 μm. The data represent the mean±s.d.; ***P<0.001, Student's *t*-test.

To examine whether FSS induces microvilli formation in placental trophoblastic cells, we cultured BeWo cells under static or fluid flow conditions using our microfluidic device. In the absence of FSS, microvilli were observed at the cell–cell contact sites, but most of the cells had sparse microvillar surfaces (Fig. 2a). In contrast, after the overnight medium perfusion in both of the channels, all cells in the maternal chamber formed microvilli over the entire cell surface (Fig. 2b,c). At the centre of the chamber, where the FSS was low (∼0.001 dyn cm^−2^), the microvillar protrusions were long (> 2 μm); however, they were shortened (<2 μm) in the area with high FSS (at the inlet or outlet of the chamber, FSS: ∼0.1 dyn cm^−2^). To quantify the microvilli formation induced by FSS, we measured total length of microvilli/field from the scanning electron microscopy (SEM) images for each FSS condition. Microvilli were increased 10.8-fold at the low-FSS area and 5.6-fold at the high-FSS area compared with the static culture conditions. The measured lengths of microvilli were significantly different between under high- and low-FSS condition (Fig. 2d).

We next examined the time course of microvilli formation to investigate the time required for microvilli induction by FSS (Fig. 2e). BeWo cells seeded in the chamber area of the device were cultured overnight under static conditions and then exposed to FSS for various durations. Microvilli formation started to be observed after 1 h of FSS exposure with numerous microvilli sprouts (Fig. 2f). FSS loading continuously increased microvilli formation over a 12-h period, but stopping the medium perfusion resulted in microvilli decrease, which indicates that FSS is required for the cells to maintain microvillar structure. We were unable to observe apparent microvilli formation when the medium was perfused only in the fetal (bottom) channel (Fig. 2e, open diamond). The formation of FSS-induced microvilli was also evident in human villous trophoblasts (HVTs) derived from the placental villi, including placental syncytiotrophoblasts. As observed in BeWo cells, most of the cells exhibited sparse microvillar surface under static conditions. After the overnight medium perfusion culture, HVTs extended the length of microvilli, whereas a small fraction of cells lacked FSS-induced microvilli (Fig. 2g–i); this variation in microvilli formation may have resulted from the contamination of non-trophoblastic cells in the primary culture.

Overall, these observations strongly indicate that FSS is a critical external cue that triggers microvilli formation in BeWo cells and HVTs.

### Facilitation of glucose transport in the FSS-exposed cells

Glucose transfer across the placental barrier is essential for placental barrier cell support of continuous embryonic growth during pregnancy^7^. The barrier cells present abundant localization of the glucose transporter type 1 (GLUT1, also known as solute carrier family 2, facilitated glucose transporter member 1: SLC2A1) at the microvillar surface, which is required for directional net transport of glucose to the fetus^8^.

**Figure 3:**
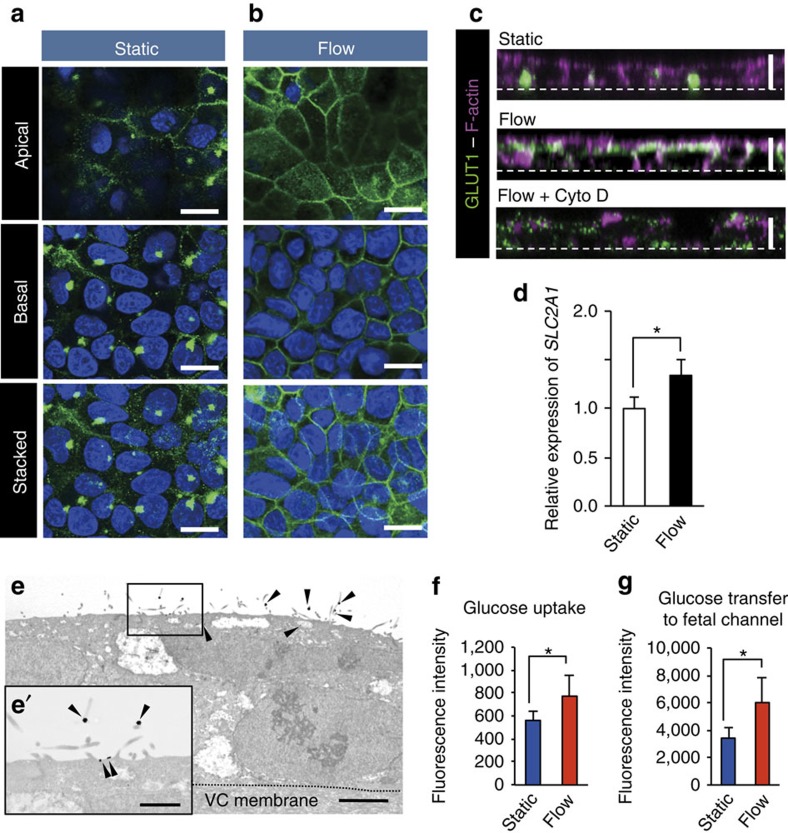
Microvillar localization of GLUT1 and facilitation of glucose transport in FSS-exposed BeWo cells. Cells were seeded in the chamber area of the device and then cultured overnight under static or fluid flow (2 μl min^−1^) conditions. (**a**,**b**) Localization of GLUT1 (green) was analysed by immunofluorescence confocal microscopy. Nuclei were counterstained with 4′,6-diamidino-2-phenylindole (DAPI; blue). Representative *x–y* optical sections at apical or basal side of cells and the stacked images were shown. Scale bar, 20 μm. (**c**) Representative confocal images of *x–z* optical sections of BeWo cells stained with GLUT1 antibody (green) and phalloidin (F-actin, magenta). The lowest panel (Flow+Cyto D) shows the image derived from cells cultured under fluid flow conditions in the presence of cytochalasin D (Cyto D, 3 μM). Scale bar, 10 μm. (**d**) Relative mRNA expression of *SLC2A1* (GLUT1) in BeWo cells cultured with or without FSS. BeWo cells were cultured overnight under static or fluid flow (2 μl min^−1^) conditions, and *SLC2A1* mRNA expression normalized to 18S ribosomal RNA level was analysed by real-time PCR. The data are presented as the mean±s.e.m. from three independent experiments. Data significance was assessed by unpaired two-tailed Student's *t*-test; *P<0.05. (**e**) The subcellular localization of GLUT1 was analysed by immunoelectron microscopy. GLUT1 signals (arrowheads) were detected at the microvillar surface of the FSS-exposed cells. The boxed area is magnified in **e′**. Scale bars, 3 μm (**e**); 1 μm (**e′**). (**f**,**g**) Facilitation of glucose uptake and transfer to the fetal channel in the FSS-exposed cells. The glucose transport activity of BeWo cells was analysed by 2-NBDG (2 mM) uptake assays. The data are presented as the mean±s.d. (*n*=5 for each condition). Significance was assessed by unpaired two-tailed Student's *t*-test; *P<0.05.

We first investigated the subcellular localization of GLUT1 in BeWo cells before and after FSS exposure (Fig. 3a–c). Under static culture conditions, GLUT1 was predominantly stained in clusters adjacent to the nuclei, which co-localized with a centriole-localizing protein (dynein, axonemal, light chain 4; DNAL4)^9^ (Fig. 3a; Supplementary Fig. 1). This localization pattern of GLUT1 was substantially different from that observed *in vivo*. In contrast, GLUT1 was localized to the apical membrane of cells and cell–cell contact region after overnight exposure of cells to FSS (Fig. 3b,c). Concurrent with the marked change of GLUT1 localization, we also observed a slight increase (1.34-fold vs static culture conditions as the control) in the messenger RNA (mRNA) expression level of the GLUT1 gene (*SLC2A1*; Fig. 3d). Treatment of BeWo cells with cytochalasin D (3 μM), an actin-depolymerization reagent, completely abolished actin assembly and localization of GLUT1 at the apical membrane of cells even under the fluid flow condition, which indicates that apical localization of GLUT1 occurs in a coupled manner with actin cytoskeletal reorganization for microvilli formation to occur (Fig. 3c).

To confirm that GLUT1 localizes in the microvillar plasma membrane, we performed immunoelectron microscopic analysis using a GLUT1 antibody. As shown in Fig. 3e, GLUT1 signals visualized using gold nanoparticles were observed at the microvillar plasma membrane in the FSS-exposed cells, which indicates that FSS-induced microvilli formation with abundant localization of GLUT1, as observed *in vivo*.

Next, we examined whether FSS affected the glucose transport activity of cells using 2-[*N*-(7-nitrobenz-2-oxa-1,3-diazol-4-yl)amino]-2-deoxy-D-glucose (2-NBDG), which is a fluorescent glucose analogue^10^,^11^. BeWo cells were cultured overnight under static or flow conditions, and then incubated with 2-NBDG (2 mM) without flow to allow glucose transfer to the fetal channel. By measuring the fluorescence in the cell lysate and fetal channel, we found that glucose uptake into the cells and subsequent transfer to the fetal channel in FSS-exposed cells were significantly increased 138% (*P*<0.05, Student's *t*-test) and 176% (*P*<0.05, Student's *t*-test), respectively (Fig. 3f,g). These data indicate that exposure of BeWo trophoblastic cells to FSS resulted in the formation of functional microvilli with the localized GLUT1 transporter. The asymmetric localization of GLUT1 may facilitate directional glucose transport in the cells.

### Activation of the TRPV6 in response to FSS

**Figure 4:**
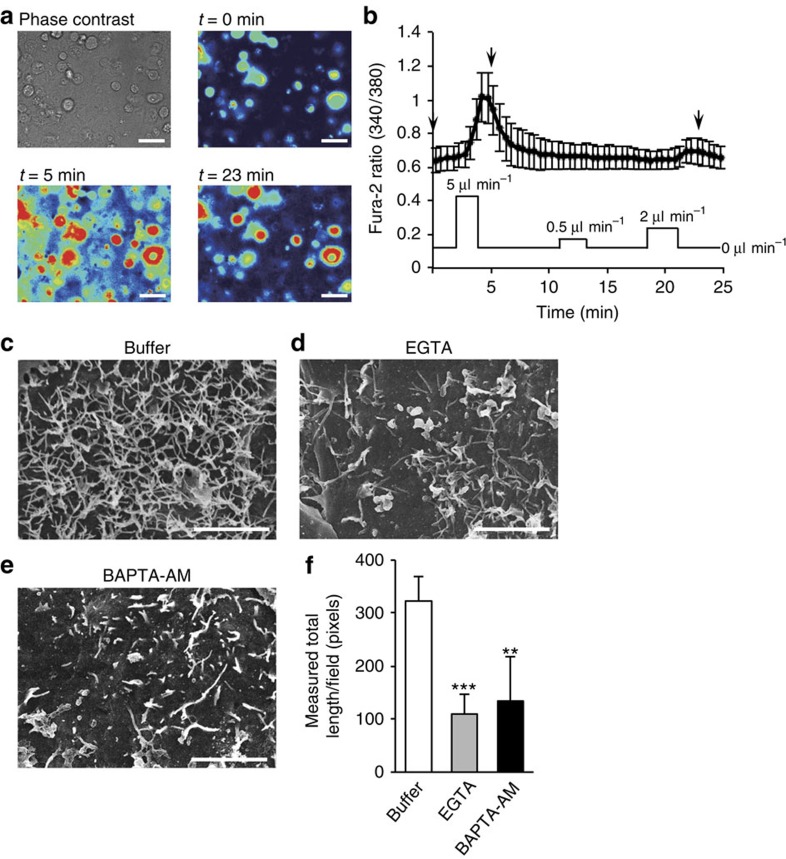
FSS induces Ca^2+^ entry and increase of intracellular [Ca^2+^] in BeWo cells. (**a**) Representative Ca^2+^ responses in BeWo cells at each indicated time point. Pseudocolored images are shown: red cells indicate high levels of intracellular [Ca^2+^] measured by the fluorescence intensity, and blue cells represent basal levels. Scale bar, 50 μm. (**b**) Time course of Fura-2 fluorescence in the BeWo cells. FSS was transiently loaded by infusing the medium at flow rates of 5 (*t*=2.0–4.2 min), 0.5 (*t*=10.9–13.6) and 2 (*t*=18.7–21.8 min) μm min^−1^. The calcium response images (**a**) were captured at the time points indicated by arrows, respectively (*t*=0, 5, 23 min). Data are shown as the Fura-2 ratio (*F*_340_/*F*_380_) and mean±s.d. (*n*=24). (**c**–**f**) Inhibition of FSS-induced microvilli formation by calcium chelators. SEM images of BeWo cells cultured under fluid flow (5 μm min^−1^) in the presence or absence of EGTA (1 mM) or BAPTA-AM (10 μM) for 3 h. The representative images (**c**–**e**) were captured at the centre area of the chamber, and total length of microvilli per field (700 μm^2^, five fields) was measured (**f**). The data represent the mean±s.d.; **P<0.01, ***P<0.001, Student's *t*-test. Scale bar, 5 μm.

To investigate the molecular mechanism underlying FSS-induced microvilli formation, we evaluated the change in the intracellular free-Ca^2+^ concentration ([Ca^2+^]), because there has been accumulating evidence that FSS triggers Ca^2+^ entry into the cells^12^,^13^,^14^,^15^. Calcium imaging using the Fura-2 calcium indicator was performed to evaluate whether FSS influences intracellular [Ca^2+^] in BeWo cells. As expected, FSS induced a gradual increase of intracellular [Ca^2+^] within a few minutes (Fig. 4a,b, *n*=24). This increase was dependent on flow rate but was undetectable at flow rate of 0.5 μl min^−1^.

To examine whether Ca^2+^ is required for FSS-induced microvilli formation in BeWo cells, culture medium that contains the calcium chelator, ethylene bis-(oxyethylenenitrilo) tetracetic acid (EGTA) or cell permeable bis-(*o*-aminophenoxy)-*N*,*N*,*N*′,*N*′-tetraacetic acid-acetoxymethyl (BAPTA-AM), was perfused for 3 h (Fig. 4c–f). Measured microvilli length in the presence of EGTA (1 mM) or BAPTA-AM (10 μM) was significantly reduced to 34.0 and 41.6% compared with that of the buffer control (Fig. 4f). These data indicate an essential role of extracellular Ca^2+^ influx in FSS-induced microvilli formation.

**Figure 5:**
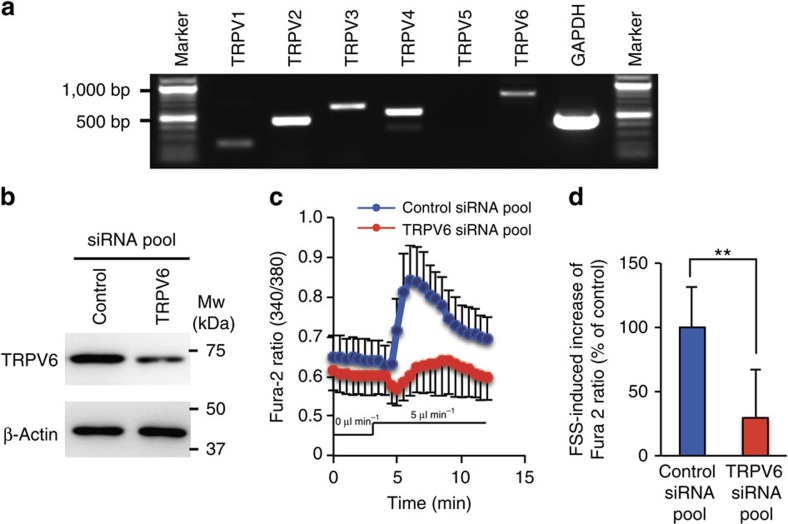
Involvement of TRPV6 in FSS-induced Ca^2+^ entry in BeWo cells. (**a**) Expression of TRPV ion channels in BeWo cells was analysed by RT–PCR. Glyceraldehyde-3-phosphate-dehydrogenase (GAPDH) was used as an internal control. (**b**) TRPV6 knockdown was evaluated by immunoblot analysis using anti-TRPV6 antibody and anti-β-actin antibody (loading control). (**c**,**d**) Time course of Fura-2 fluorescence in the TRPV6 knockdown BeWo cells. Fluid flow (5 μl min^−1^) was applied from *t*=3 min onward, and calcium imaging was performed at the centre area of the chamber. Data shown represent the Fura-2 ratio (*F*_340_/*F*_380_) and mean±s.d. (*n*=33). The FSS-induced increase of the Fura-2 ratio (maximal Fura-2 ratio−basal Fura-2 ratio (*t*=3 min)) in **c** is shown as the percentage relative to the control (**d**). **P<0.01, Student's *t*-test.

Microvilli contain various mechanosensitive cation channels including TRP protein family, which are activated by a wide range of physciochemical stimuli^16^,^17^. Although some subfamilies of TRP channels are capable of a transduction of mechanical signals into the increase of cytosolic calcium, it has been reported that TRPV5 and TRPV6, both of which are TRPV subfamily members, are Ca^2+^-selective ion channels that play a pivotal role in the calcium absorption in epithelial tissues, including the intestine, kidney and placenta^18^,^19^,^20^. In our preliminary experiment, capsaicin, which is known to activate TRPV channels^21^,^22^, induced microvilli in the immunocytochemical detection of Ezrin, as well as *Ezrin* mRNA levels (approximately twofold at 1 μM capsaicin compared with the control) in BeWo cells cultured under static conditions (Supplementary Fig. 2; Ezrin is a microvilli-localizing protein that functions as a linker between plasma membrane and microvillous actin cytoskeleton^23^). Moreover, addition of a general inhibitor of receptor-operated calcium entry, SKF96365, resulted in the partial inhibition of microvilli formation visualized by Ezrin (Supplementary Fig. 3). On the basis of these facts, we first investigated the expression of TRPV subfamily, which consists of six homologous members, in BeWo cells by reverse transcription PCR (RT–PCR; Fig. 5a). BeWo cells expressed *TRPV1*, *TRPV2*, *TRPV3*, *TRPV4* and *TRPV6*. Above all, TRPV6 was reported to be activated by FSS^24^, which indicates that TRPV6 is a candidate responsible for FSS-induced Ca^2+^ influx in BeWo cells.

We used short interfering RNA (siRNA) TRPV6 knockdown and loaded FSS on the cells. Transfection of TRPV6 siRNA resulted in marked reduction of TRPV6 protein level at 24–48 h after transfection compared with transfection of the control siRNA (Fig. 5b). As shown in Fig. 5c,d, we observed that cells with TRPV6 knockdown exhibited delayed calcium response and poor increase of FSS-induced intracellular [Ca^2+^], with a reduction of ∼70% relative to the control group (*n*=33, *P*<0.01, Student's *t*-test). There was no significant effect of TRPV6 knockdown on cell viability (Supplementary Fig. 4). These results clearly indicate that TRPV6 plays a critical role in FSS-induced Ca^2+^ influx and intracellular [Ca^2+^] increase in BeWo cells.

### Downstream phosphorylation signalling of TRPV6

**Figure 6:**
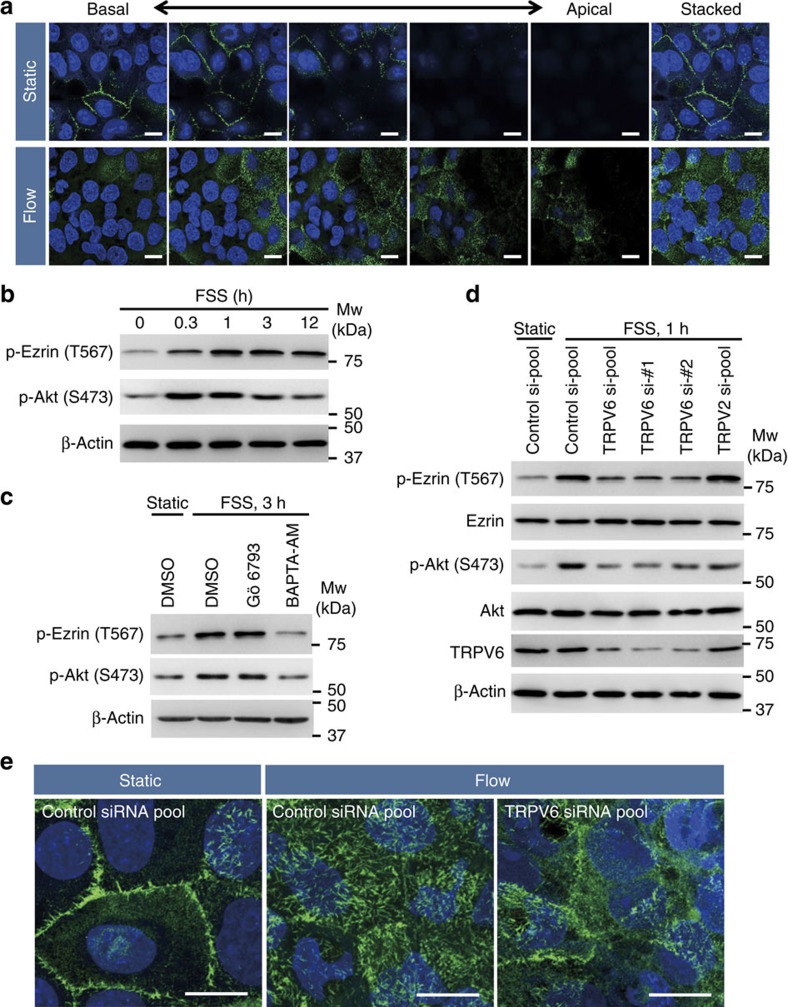
Essential roles of TRPV6 in FSS-induced microvilli formation. (**a**) Apical localization of ezrin in the FSS-exposed BeWo cells. Cells were cultured overnight under static conditions, and then cultured with or without medium perfusion (5 μl min^−1^) for 1 h. Ezrin (green) localization was observed by immunofluorescence confocal microscopy. Nuclei were counterstained with 4′,6-diamidino-2-phenylindole (DAPI; blue). Serial *x–y* focal planes (z-sections, 0.88 μm interval in ‘Static', 0.90 μm in ‘Flow') and the stacked images are shown. Scale bar, 20 μm. (**b**) Time-course phosphorylation of Ezrin (pThr567) and Akt (pSer473) in response to FSS. BeWo cells were cultured overnight under static conditions and exposed to FSS (5 μl min^−1^) for the indicated times. The cells were lysed, and protein expression level or phosphorylation was analysed by immunoblotting. (**c**) Inhibition of Akt phosphorylation by BAPTA-AM. Gö6793 (100 nM), BAPTA-AM (10 μM) or buffer control (DMSO) was added to the perfusing medium. (**d**) Involvement of TRPV6 in the FSS-induced Ezrin phosphorylation. BeWo cells transfected with siRNA were seeded in the chamber area of the device and cultured overnight under static conditions before exposure to FSS for 1 h. β-Actin was used as a loading control. Note that the TRPV6 siRNA oligo (TRPV6 si-#1 or #2), as well as the siRNA pool, shows similar inhibitory effects on the FSS-induced phosphorylation of Ezrin and Akt. (**e**) Impaired Ezrin localization in the TRPV6 knockdown cells. Ezrin localization in the TRPV6 knockdown cells was analysed by immunofluorescence confocal microscopy. Note that TRPV6 knockdown cells exposed to FSS (5 μl min^−1^) for 1 h failed to show the microvillous localization pattern of Ezrin as observed in the control siRNA cells under FSS. Scale bar, 20 μm.

In our device, we observed remarkable change of Ezrin localization after 1 h of FSS exposure (Fig. 6a), at which point numerous microvillar sprouts were observed under SEM observation (Fig. 2f). Under static culture conditions, Ezrin was localized in the cell–cell contact sites, where the presence of microvilli was evident based on SEM observation. Meanwhile, in the FSS-exposed cells, Ezrin was predominantly detected at the apical membrane of cells (Fig. 6a). Ezrin expression in mRNA or protein level did not significantly change after overnight exposure to FSS (Supplementary Fig. 5). These data indicate that not the upregulation of *Ezrin* mRNA, but relocalization of Ezrin protein to the apical membrane of cells is an initial step for FSS-induced microvilli formation in BeWo cells.

To identify the signalling molecule underlying FSS-induced microvilli formation, we first examined Ezrin phosphorylation at Thr567 that is required to restrict the function of Ezrin to the apical membrane^25^,^26^,^27^. Consistent with the time course of microvilli formation, Ezrin phosphorylation was detected after 20 min of FSS exposure and reached its maximal level after 1 h of FSS exposure (Fig. 6b).

We next analysed the upstream signalling molecules involved in the regulation of Ezrin phosphorylation on FSS. Because previous studies reported that Ezrin phosphorylation at Thr567 is regulated via Akt, p38 mitogen-activated protein kinase or protein kinase C signalling^28^,^29^,^30^,^31^,^32^, we examined whether these kinases are phosphorylated on FSS or involved in ezrin phosphorylation using their pharmacological inhibitors. We found that phosphorylation of Akt at Ser473 was induced in response to FSS (Fig. 6b), and the addition of the Akt kinase inhibitor (Akt inhibitor VIII trifluoroacetate salt hydrate) in the perfusing medium suppressed FSS-induced Ezrin phosphorylation (Supplementary Fig. 6). We detected a slight increase in p38 mitogen-activated protein kinase phosphorylation on FSS, but its inhibitor (SB203580) had no effect on FSS-induced Ezrin phosphorylation (Supplementary Fig. 6). Moreover, treatment of cells with BAPTA-AM markedly inhibited the FSS-induced Akt phosphorylation, as well as the Ezrin phosphorylation, whereas Gö6793, a protein kinase C inhibitor, had no effect (Fig. 6c). These results indicate that Ezrin phosphorylation at Thr567 on FSS is regulated via Ca^2+^-dependent phosphorylation of Akt.

Finally, we examined whether this phosphorylation signalling is downstream of TRPV6 by siRNA TRPV6 knockdown. As shown in Fig. 6d, phosphorylation of Ezrin and Akt was markedly reduced by siRNA TRPV6 knockdown, whereas transfection of TRPV2 siRNA (the knockdown efficiency was ∼90%, Supplementary Fig. 7) had no effect on Ezrin phosphorylation, indicating that FSS-induced Akt-Ezrin phosphorylation signalling is a downstream event of TRPV6. There was no significant decrease in the total protein level of Ezrin or Akt by TRPV6 knockdown. Consistent with the reduced phosphorylation level, microvillar localization of Ezrin under FSS was disturbed in the TRPV6 knockdown cells compared with the FSS-exposed cells transfected with control siRNA (Fig. 6e).

## Discussion

In this study, using a microfluidic device and trophoblastic cells, we demonstrated that FSS is an extracellular cue that triggers microvilli formation. Calcium-imaging experiments showed that FSS-induced Ca^2+^ influx and intracellular [Ca^2+^] increase were essential for microvilli formation. Silencing of TRPV6 further revealed that TRPV6 ion channel opens in response to FSS and regulates Ezrin phosphorylation at Thr567 via Akt in a Ca^2+^-dependent manner. This phosphorylation signalling was required for the microvillar localization of Ezrin. These data strongly indicate that FSS triggers Ca^2+^ entry via mechanosensitive activation of TRPV6, which regulates microvilli formation through the functional activation of Ezrin via Ca^2+^-dependent Akt phosphorylation.

Microvilli are recognized as intrinsic structures of differentiated cells, therefore, previous studies were focused on genetic approaches to understand the molecular basis of microvilli formation; gain-of-function and loss-of-function gene analyses identified a set of genes involved in the microvilli formation^5^,^6^,^25^,^33^,^34^. Here we found that FSS, an external mechanical cue, was a trigger for microvilli formation. In our system, BeWo cells were exposed to a low range of FSS (0.001–0.1 dyn cm^−2^) relative to the typical FSS for blood vessels (1–10 dyn cm^−2^). This range is considered to reflect the physiological FSS of placental barrier cells because they are located in the wide cavity of intervillous space where the flow rate of the maternal blood is drastically reduced^35^. Indeed, under such a low-level FSS, HVTs derived from the human placental villi of the placental barrier similarly formed microvilli, which indicates that FSS-induced microvilli formation in the present study is physiologically relevant. Moreover, within this range of FSS, microvilli length markedly changed depending on FSS magnitude; shorter microvilli were observed with higher FSS and longer microvilli were observed with lower FSS. It therefore seems reasonable that the cells effectively absorb nutrients in the intervillous space where the barrier cells are exposed to various degrees of FSS depending on the distance from the spiral arteries or the pregnancy duration^36^.

Our study highlighted a crucial role for TRPV6, a Ca^2+^-selective ion channel that belongs to TRP channel superfamily, in FSS-induced microvilli formation. Although TRP channels are known to be activated by various extracellular stimuli^37^,^38^, little is known about TRPV6 activation. In the present study, through calcium imaging and TRPV6 knockdown in BeWo cells, we first showed that TRPV6-mediated Ca^2+^ influx in the cells were induced by fluid flow in an FSS magnitude-dependent manner. This finding is consistent with that of a recent study, which showed that TRPV6 expressed in HEK293 cells is still activated by fluid flow^24^. Our results thus indicate that TRPV6 activation is mechanosensitively regulated in trophoblastic cells.

TRPV6 is predominantly expressed in epithelial tissues, such as the kidney, intestine and placental syncytium; thus, it is considered physiologically important as a Ca^2+^ transport channel to maintain calcium homeostasis in the body^18^,^19^,^39^,^40^,^41^. Therefore, most studies on TRPV6 have addressed transcellular transport of Ca^2+^. Here we found that TRPV6 mediates Akt-Ezrin phosphorylation signalling necessary for FSS-induced microvilli formation. Although TRPV6 expression in BeWo cells is relatively low compared with TRPV2 that is the most highly expressed mechanoresponsive TRPV channel, siRNA TRPV6 knockdown profoundly abolished FSS-induced Ezrin phosphorylation. This result is probably due to the difference in Ca^2+^ permeability between TRPV2 and TRPV6; it was reported that TRPV6 is more permeable to Ca^2+^ (Ca^2+^/Na^+^>100) than TRPV2 (Ca^2+^/Na^+^=1–10)^42^. We also found that BeWo cells express TRPV4 channel that has been reported to activate by FSS. However, microvilli formation is not likely to involve TRPV4 activation because of the requirement of higher range of FSS for its activation (>3 dyne cm^−2^)^43^. Indeed, our data on Ca^2+^ imaging demonstrated that TRPV6 was responsible for FSS-induced Ca^2+^ influx in BeWo cells. These data indicate a pivotal role of TRPV6 in the FSS-induced microvilli formation.

On the basis of the inhibitory effect of calcium chelators and TRPV6 siRNA on microvilli formation, we showed that FSS triggers TRPV6-mediated Ca^2+^ influx and intracellular calcium signalling that regulates apical localization of Ezrin. It is reasonable to consider that FSS-induced microvilli formation is regulated through phosphorylation signalling rather than gene transcription because microvilli were observed within 1 h of FSS exposure with numerous microvilli sprouts. Indeed, Ezrin mRNA expression and protein level were not significantly increased after overnight culture with fluid flow, whereas Ezrin localization remarkably changed from the cell–cell contact region to the apical membrane of cells within 1 h of FSS exposure. Concomitant with the relocalization, Ezrin was phosphorylated at Thr567 in parallel with Akt kinase activation. The rapid phosphorylation signalling mediated by the mechanosensitively regulated TRPV6 may benefit placental barrier functions of cells exposed to the dynamic fluid environment. Since it is still uncertain whether Ezrin is directly phosphorylated by Akt, more detailed analysis of the downstream calcium signalling pathway, including calmodulin, calcium/calmodulin-dependent protein kinase (CaMK) kinase and CaMKII, is important to elucidate the molecular mechanism of FSS-induced microvilli formation.

Recently, Ingber *et al.* proposed a series of biomimetic microsystems called ‘organ-on-a-chip platform'^44^,^45^,^46^. These studies demonstrated the reconstitution of multiple organ-level functions on a microfabricated chip through control of tissue geometry, cell–cell or cell–extracellular matrix interactions in addition to mechanotransduction. In our system, by loading FSS and utilizing a vitrified collagen (VC) cell scaffold, we successfully reconstituted the microvillar surface of the human placental barrier with apically localized GLUT1 membrane transporters, as observed *in vivo*. Noticeably, although FSS-induced microvilli formation was also observed on the porous membrane support that has been used in organ-on-a-chip studies (Supplementary Fig. 8), we detected a significant increase in glucose transport in FSS-exposed cells cultured on the VC membrane but not on the porous membrane support (Supplementary Fig. 9). This difference in transport activity probably resulted from the partial loss of cell polarity on the porous scaffold, which indicates that cell scaffold may affect the polarized localization of membrane transporters and thus affect the directional transport activity of FSS-exposed trophoblastic cells. The human placental barrier exhibits polarized localization of several drug transporters, including multidrug resistance proteins and multidrug resistance-associated proteins^47^; therefore, our microfluidic system with the VC membrane appears to be advantageous for analysing placental drug transport *in vitro*, whereas conventional static analytical systems with transwell chambers poorly form both the microvillar surface and apical–basolateral polarity^48^. In addition, interspecies differences in the placental structure and expression of membrane transporters make it difficult to obtain a representative *in vivo* model of the human placental barrier^49^,^50^. Hence, our microfluidic system may serve as a powerful tool to study the molecular basis of the directional transport activity of the human placental barrier in addition to pharmacokinetics during pregnancy.

## Methods

### Device design and fabrication

The microfluidic device is composed of two microfluidic channels mimicking a maternal (upper layer) and a fetal (bottom layer) blood circulation, separated by a VC membrane (10-μm thickness, Asahi Techno Glass, Shizuoka, Japan). The cross-sectional size of the microchannels was 2 mm (width) × 200 μm (height), and the channel length was 20 mm for the fetal channel and 15 mm for the maternal channel. The microfluidic channels were prepared by replica mounting in polydimethylsiloxane (PDMS; SYLPOT 184, Dow Corning Toray, Tokyo, Japan) with a 10:1 (w/w) ratio of crosslinking agent, using a SU-8 (SU-8 100, MicroChem Corp., MA) master fabricated by standard photolithographic methods. After thermal curing of the patterned microchannels, the maternal chamber structure was introduced at the centre of the maternal channel using a biopsy punch (*φ*=4 mm). The VC membrane (7 × 7-mm pieces) was then assembled with fetal and maternal PDMS channels as to completely cover the maternal chamber. Both of the PDMS layers were O_2_ plasma (SAMCO) treated and incubated for 90 min at 75 °C to facilitate permanent bonding.

### Cell culture and flow experiments

BeWo trophoblastic cells, which were established from a human choriocarcinoma^48^,^51^, were provided by the RIKEN BRC through the National Bio-Resource Project of MEXT, Saitama, Japan. The cells were cultured in Ham's F12 Nutrient Mixture (Sigma, St Louis, MO) supplemented with 10% fetal bovine serum and 50 μg ml^−1^ kanamycin sulfate (Sigma). HVTs (ScienCell, Carlsbad, CA) were maintained in trophoblast medium supplemented with fetal bovine serum and trophoblast growth factors (ScienCell) and used for experiments at passage 3. Before seeding on the device, the microchannels were filled with culture medium to rehydrate the VC membrane for 30 min. BeWo cells were collected using 0.05% Trypsin/EDTA, and 20 μl of cell suspension (3.0 × 10^4^ cells) was added to the maternal chamber. The maternal chamber was then sealed with a thin PDMS membrane, and the device was incubated in a CO_2_ incubator to allow cell adhesion on the VC membrane. After 1–2 h of culture, the device was connected to a peristaltic pump (GILSON, Middleton, WI) to control the flow rate. All cell cultures were performed at 37 °C in a humidified incubator with 5% CO_2_. On the basis of the computational simulation of shear velocity using COMSOL Multiphysics (COMSOL AB, Stockholm, Sweden) (Supplementary Fig. 10), the FSS in the maternal chamber was estimated to range from 0.001 (at the centre of the chamber; low FSS) to 0.12 dyn cm^−2^ (at the inlet or the outlet area of chamber; high FSS; when the flow rate is 5 μl min^−1^), following the equation: *τ*=*μ* × *δu*/*δy*, where *μ* is the viscosity of the cell culture medium with a viscosity value of 9.5 × 10^−4^ Pa·s at 37 °C, and *δu*/*δy* is the shear velocity. We confirmed that overnight culture under static conditions in our device did not cause serious starvation of oxygen or nutrients in BeWo cells (Supplementary Fig. 11).

### RNA interference

BeWo cells were grown to 50% confluence and incubated with DharmaFect1 transfection reagent (2 μl in 1 ml of culture medium) complexed with 25 nM siGENOME SMARTpool against human TRPV6 (5′-GGAAACAGCGCUACACAUA-3′, 5′-GGACAAAGACUCAGUGGAA-3′, 5′-UGAACAAGUUGCUCAAGUA-3′, 5′-CCAUAUAUCUGCUGUACAU-3′), TRPV2 (5′-GGUAAGACGUGCCUGAUGA-3′, 5′-GAAAUGGGAUCUGCUCAUC-3′, 5′-GGCUGAACCUGCUUUACUA-3′, 5′-GCGAGACCGUCAACAGUGU-3′) or 25 nM siGENOME Non-Targeting siRNA Pool#1 (5′-UAGCGACUAAACACAUCAA-3′, 5′-UAAGGCUAUGAAGAGAUAC-3′, 5′-AUGUAUUGGCCUGUAUUAG-3′, 5′-AUGAACGUGAAUUGCUCAA-3′) (Dharmacon, Thermo Scientific, Lafayette, CO, USA). In some experiments, TRPV6 siRNA oligo against a single target sequence (#1: 5′-GGACAAAGACUCAGUGGAA-3′, #2: 5′-UGAACAAGUUGCUCAAGUA-3′, respectively) was used to confirm specificity of siRNA effect. TRPV6 siRNA oligos were synthesized by Sigma-Aldrich (St Louis, MO, USA). After 7–12 h, the transfection medium was replaced with fresh medium. The siRNA-transfected cells were cultured overnight and then seeded onto the device.

### Immunofluorescence

BeWo cells were seeded in the device at 3.0 × 10^4^ cells per maternal chamber and cultured overnight with or without medium perfusion. In some experiments, BeWo cells grown to subconfluence on a type-I collagen-coated glass-bottom dish (IWAKI, Tokyo, Japan) were incubated overnight with capsaicin (Sigma). The cells were rinsed with PBS and fixed with 4% paraformaldehyde in PBS (4% PFA/PBS) for 20 min at room temperature. After fixation, cells were permeabilized with 0.2 or 0.01% Triton X-100 for 5–10 min and incubated with 5% skim milk (BD Difco, Sparks, MD, UAS) in PBS for 20 min to block non-specific binding of the antibody. These preparations were then incubated at 4 °C overnight with the primary antibodies: mouse anti-ezrin monoclonal antibody (1:300, ab4069, Abcam, Cambridge, MA, USA), mouse anti-glucose transporter GLUT1 monoclonal antibody (1:300, clone SPM498, ab40084, Abcam) or AlexaFluor 568-conjugated phalloidin (1:300, A12380, Molecular Probes, Eugene, OR, USA). The primary antibody incubation was followed by 1 h of incubation with Alexa Fluor488-conjugated goat anti-mouse IgG secondary antibody (1:300, A11017, Molecular Probes) and 1 μg ml^−1^ 4′,6-diamidino-2-phenylindole (Sigma) in 5% skim milk/PBS. Stained samples were mounted in Fluoromount/Plus medium (Diagnostic BioSystems, Pleasanton, CA, USA) to suppress photobleaching. Cross-sectional and stacked images were acquired by confocal laser scanning microscopy LSM780 and ZEN imaging software (Carl Zeiss Microscopy, Jena, Germany).

### RT–PCR and TaqMan gene expression analysis

Total RNA was prepared using a PureLink RNA Mini Kit (Life Technologies, Carlsbad, CA, USA). First strand complementary DNA was synthesized from 1 μg (for RT–PCR) or 100 ng (for TaqMan assay) of total RNA using SuperScript III reverse transcriptase (Life Technologies) and Oligo-dT primers (Life Technologies) for RT–PCR or random hexamers (New England BioLabs Inc., Ipswich, MA, USA) for the TaqMan assay. The primer sequences used for the RT–PCR analyses were as follows: human glyceraldehyde-3-phosphate-dehydrogenase forward primer: 5′-ACCACAGTCCATGCCATCAC-3′; human glyceraldehyde-3-phosphate-dehydrogenase reverse primer: 5′-TCCACCACCCTGTTGCTGTA-3′; human TRPV6 forward primer: 5′-CTCTGCCTATGGAGCAAGTTCTGC-3′; human TRPV6 reverse primer: 5′-GAGAGTCGAGGTCAGTGGTCC-3′. Primer pairs used for human TRPV1, TRPV2, TRPV3, TRPV4 and TRPV5 were as previously reported^52^. The original image of DNA gel electrophoresis is shown in Supplementary Fig. 12. Quantitative real-time RT–PCR was performed with the StepOne Real-Time PCR system (Applied Biosystems/Life Technologies) using TaqMan Fast Advanced Master Mix and TaqMan Gene Expression Assays (*SLC2A1*, Hs00892681_m1 and *RN18S1*, Hs03928985_g1 as an internal control) according to the manufacturer's instructions. Reactions were run in triplicate and analysed by the comparative *C*_T_ method.

### 2-NBDG uptake and transport to the fetal channel

BeWo cells (3.0 × 10^4^ cells per maternal chamber) were seeded on the device and cultured for 20 h with or without medium perfusion (2 μl min^−1^). Fetal and maternal channels were washed with balanced salt solution (BSS; 136 mM NaCl, 5 mM KCl, 1 mM CaCl_2_, 1 mM MgCl_2_ and 18 mM HEPES, pH 7.4), and the maternal channel including the chamber part was gently perfused with 2 mM 2-NBDG(Peptide Institute, Osaka, Japan). The device was then incubated in a CO_2_ incubator for 20 min without fluid flow to enable glucose uptake in the cells. After the incubation, the BSS buffer in the fetal channel was recovered and diluted to a volume of 100 μl with BSS buffer. The fluorescence in this buffer was measured to determine glucose transfer to the fetal channel. To quantify the 2-NBDG uptakes into BeWo cells, the cells were thoroughly washed with BSS and lysed with lysis buffer (1% TritonX-100, 50 mM Tris-HCl (pH8.0), 150 mM NaCl and 1 mM EDTA). The lysate was recovered and diluted to 50 μl with the lysis buffer. Fluorescence was measured by a Nanodrop 3300 Fluorospectrometer (Thermo Scientific, Waltman, MA, USA).

### Scanning electron microscopy

BeWo cells (3.0 × 10^4^ cells per maternal chamber) were seeded on the device, cultured for 20 h with or without medium perfusion (5 μl min^−1^) and then fixed with 2% glutaraldehyde (NACALAI TESQUE, Kyoto, Japan) for 1 h at room temperature. In some experiments, the cells were cultured with medium perfusion in the presence of the calcium chelators, EGTA (Sigma) and BAPTA-AM (Sigma). The fixed samples were dehydrated with a series of dilutions of ethanol (Wako Pure Chemicals, Tokyo, Japan), substituted with *t*-butyl alcohol (Wako), freeze dried and subjected to osmium conductive metal coating by a Plasma Coater HPC-30 W (Shinku Device, Mito, Japan). SEM observation was performed using a FE-SEM (field emission scanning electron microscopy) SU8000 (Hitachi, Tokyo, Japan) at 5 kV.

### Measurement of the length of microvilli

SEM images of microvilli on the surface of cells were captured with an FE-SEM SU8000 (Hitachi) at 5 kV. The morphological analysis was performed using ImageJ 1.48v and the Analyze Skelton plugin^53^. To highlight microvilli signals, we first applied the intensity threshold to individual images. Then, the framework images of microvilli were created using the Skeletonize command. The distance between individual end points and branch points of skeletonized microvilli was calculated using Analyze Skelton (2D/3D). To avoid overestimation, we adopted the minimal branch length as the minimal-estimated microvilli length. Since, in the low-FSS area, abundant microvilli formation was observed, it was difficult to measure the length of an individual microvillus. Thus, the total length of microvilli/field was calculated by summation of the individual microvilli length.

### Immunoelectron microscopy

Cells were immunostained with mouse anti-GLUT1 monoclonal antibody (1:300, clone SPM498, ab40084, Abcam) and anti-mouse IgG conjugated with nanogold particles (1:50, provided by Tokai Electron Microscopy, Nagoya, Japan), and then fixed with 2% glutaraldehyde in 0.1 M phosphate buffer (pH 7.4) at 4 °C overnight. Preparation of the ultrathin sections and imaging were performed by Tokai Electron Microscopy. In brief, the fixed samples were processed with GoldEnhance EM Formulation (Nanoprobes, Yaphank, NY, USA) to enhance the gold particle signals and post-fixed with 2% osmium tetroxide at 4 °C overnight. After dehydration with a series of ethanol dilutions, the samples were infiltrated with propylene oxide, embedded in a resin (Quetol-812, Nisshin EM, Tokyo, Japan). The blocks were ultrathin sectioned at 90 nm with a diamond knife using an ultramicrotome (Ultracut UCT, Leica, Mannheim, Germany), and stained with uranyl acetate followed by lead stain solution (Sigma-Aldrich). The samples were observed under transmission electron microscopy (JEM-1400Plus, JEOL Ltd, Tokyo, Japan) at an acceleration voltage of 80 kV. Digital images were taken with a charge-coupled device camera (Veleta, Olympus Soft Imaging Solutions GmbH, Münster, Germany).

### Ca^2+^ imaging

Ca^2+^ imaging was performed with the maternal PDMS channel bonded with a glass substrate (type-I collagen coated) instead of the multilayer PDMS microchannel to avoid autofluorescence from the VC membrane. BeWo cells (3.0 × 10^4^ cells per maternal chamber) were seeded on the device and allowed to adhere for 3–6 h at 37 °C, incubated for 1 h with 2 μM Fura-2-AM calcium indicator (Life Technologies) and gently washed three times with fresh medium. The fluorescence images were captured under an inverted microscope (IX71, Olympus, Tokyo, Japan). The time course of the fluorescence intensity in the cells was monitored using Aquacosmos software and a CCD camera (ORCA-R2, Hamamatsu Photonics, Shizuoka, Japan).

### Immunoblotting

The cells cultured in the chamber area of the device were lysed with 100 μl of 1 × SDS sample buffer (50 mM Tris-HCl (pH 6.8), 2% SDS, 50 mM dithiothreitol, and 10% glycerol). The samples were sonicated, boiled, resolved by SDS–polyacrylamide gel electrophoresis using 10% acrylamide gel and transferred onto nitrocellulose membrane (Bio-Rad, Hercules, CA, USA). Membranes were preincubated with 3.2% skim milk in Tris-buffered saline (20 mM Tris, pH 7.4 and 150 mM NaCl) for 30 min and incubated at 4 °C overnight with primary antibody, which included mouse anti-Ezrin monoclonal antibody (1:200, ab4069, Abcam), rabbit anti-Ezrin (phospho-T567) polyclonal antibody (1:1,000, ab47293, Abcam), rabbit anti-Akt antibody (1:1,000, #9272, Cell Signaling Technology, Beverly, MA, UAS), rabbit anti-phospho-Akt (Ser473) monoclonal antibody (1:1,000, #4060, Cell Signaling Technology), rabbit anti-TRPV6 polyclonal antibody (1:100, sc-28763, Santa Cruz Biotech, Santa Cruz, CA, USA) or mouse anti-β-actin monoclonal antibody (1:5,000, clone AC15, A1978, Sigma-Aldrich). Blots were washed with Tris-buffered saline containing 0.05% Tween 20, and incubated with horseradish peroxidase-conjugated anti-mouse (1:5,000, NA9310, GE Healthcare Bio-Sciences, Uppsala, Sweden) or anti-rabbit (1:5,000, NA9340, GE Healthcare) IgG secondary antibody. Antibody detection was performed using ImmunoStar Zeta (Wako). The original images of immunoblots are shown in Supplementary Fig. 13.

## Additional information

**How to cite this article:** Miura, S. *et al.* Fluid shear triggers microvilli formation via mechanosensitive activation of TRPV6. *Nat. Commun.* 6:8871 doi: 10.1038/ncomms9871 (2015).

## Supplementary Material

Supplementary Information Supplementary Figures 1-13 and Supplementary Methods

## Figures and Tables

**Figure 1:**
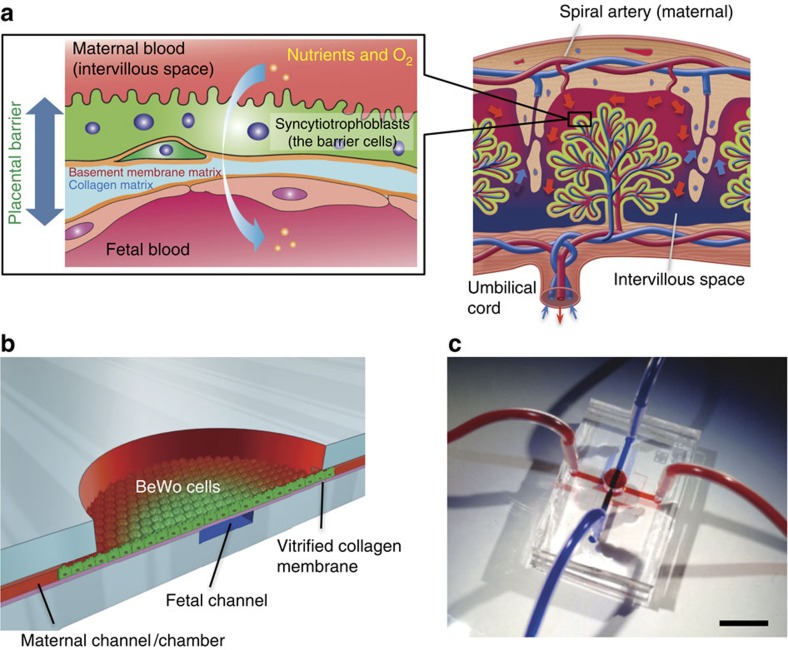
Microfluidic device for placental transfer analysis. (**a**) Schematic representation of the human placental barrier. In the placenta, maternal blood comes from the spiral artery and flows into intervillous space, into which placental villi carrying fetal blood capillaries project. Syncytiotrophoblasts, the placental barrier cells that cover the placental villi, develop a microvillar surface and function as a permeable barrier between maternal and fetal blood circulation. (**b**) Design of the microfluidic device for human placental transfer. PDMS microchannels (width, 1 mm; height, 200 μm) that correspond to maternal and fetal blood circulation are assembled with a vitrified collagen (VC) membrane and covalently bonded by O_2_ plasma treatment. The maternal microchannel has a chamber structure (*φ*=4 mm) that mimics the wide blood space of the intervillous space. (**c**) Fabricated PDMS device. Maternal and fetal channels were visualized by infusing red (maternal) and blue (fetal) ink. The material transfer between the microchannels was designed to only occur through the cell layer cultured on the VC membrane. Scale bar, 1 cm.
