# Supplementary material for: Fluid shear triggers microvilli formation via mechanosensitive activation of TRPV6
Source: Nat Commun. 2015 Nov 13;6:8871. doi: 10.1038/ncomms9871 (PMC4660203; doi:10.1038/ncomms9871)
Supplement: Supplementary Information — Supplementary Figures 1-13 and Supplementary Methods [file ncomms9871-s1.pdf]

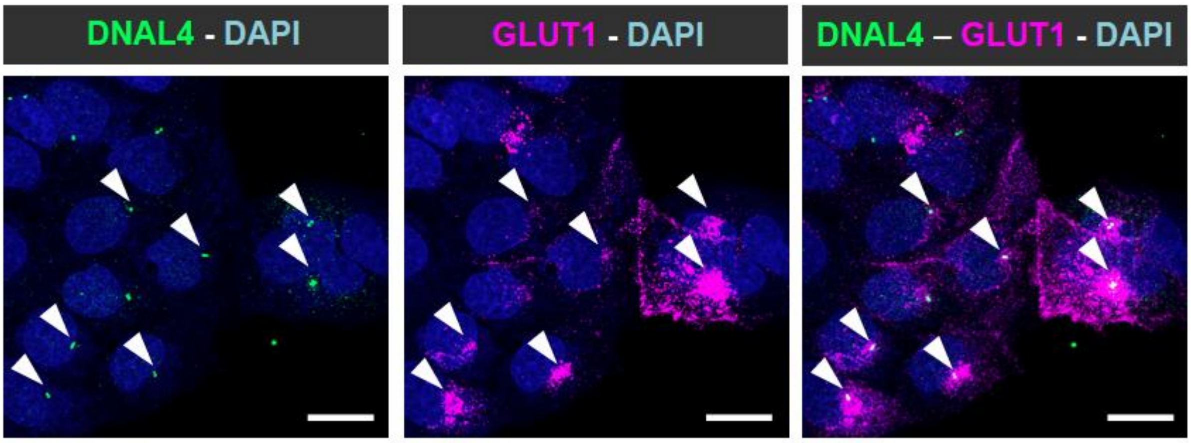

Supplementary Figure 1. Co-localization of GLUT1 and DNAL4 in BeWo cells cultured under static conditions. Cells were seeded in the chamber area of the device and cultured overnight without medium perfusion. Fixed cells were immunostained with GLUT1 (*magenta*) and DNAL4 (dynein, axonemal, light chain 4; *green*) antibody. Nuclei were counterstained with DAPI. Note that most of the clustered GLUT1 signals were co-localized with the centriole marker, DNAL4 (arrowheads). Scale bars, 20 μm.

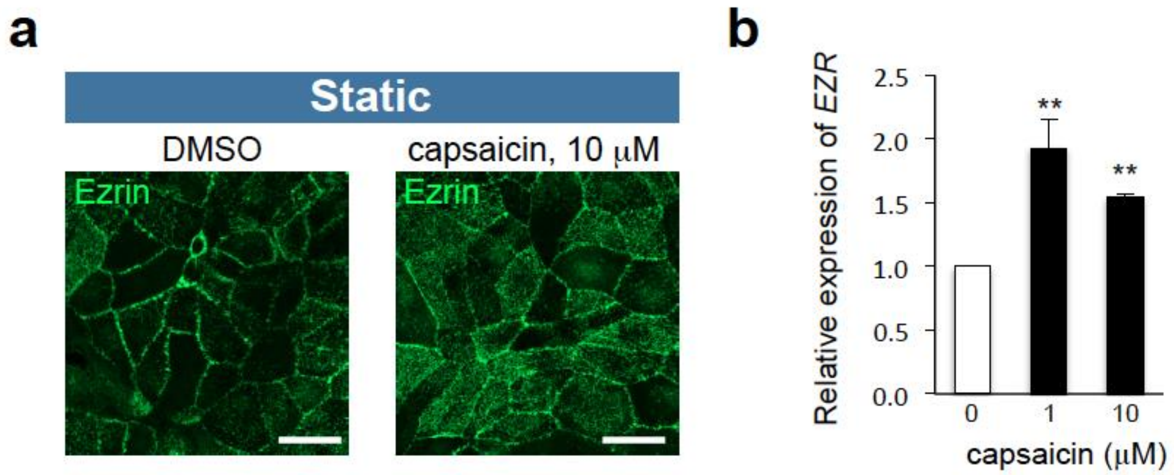

Supplementary Figure 2. Stimulatory effects of capsaicin on Ezrin re-localization and mRNA expression. BeWo cells were cultured overnight under static conditions in the presence or absence of capsaicin. Subcellular localization of Ezrin and the mRNA expression levels were analyzed by immunofluorescence (**a**) or quantitative real-time PCR (**b**). Relative mRNA expression of *EZR* is normalized to the corresponding of 18S ribosomal RNA. Data are shown as the mean ± s.e.m from three independent experiments in a triplicate assay. Data significance was assessed by unpaired two-tailed Student's *t*-test; $^{**}P < 0.01$. Bars, 50 μm.

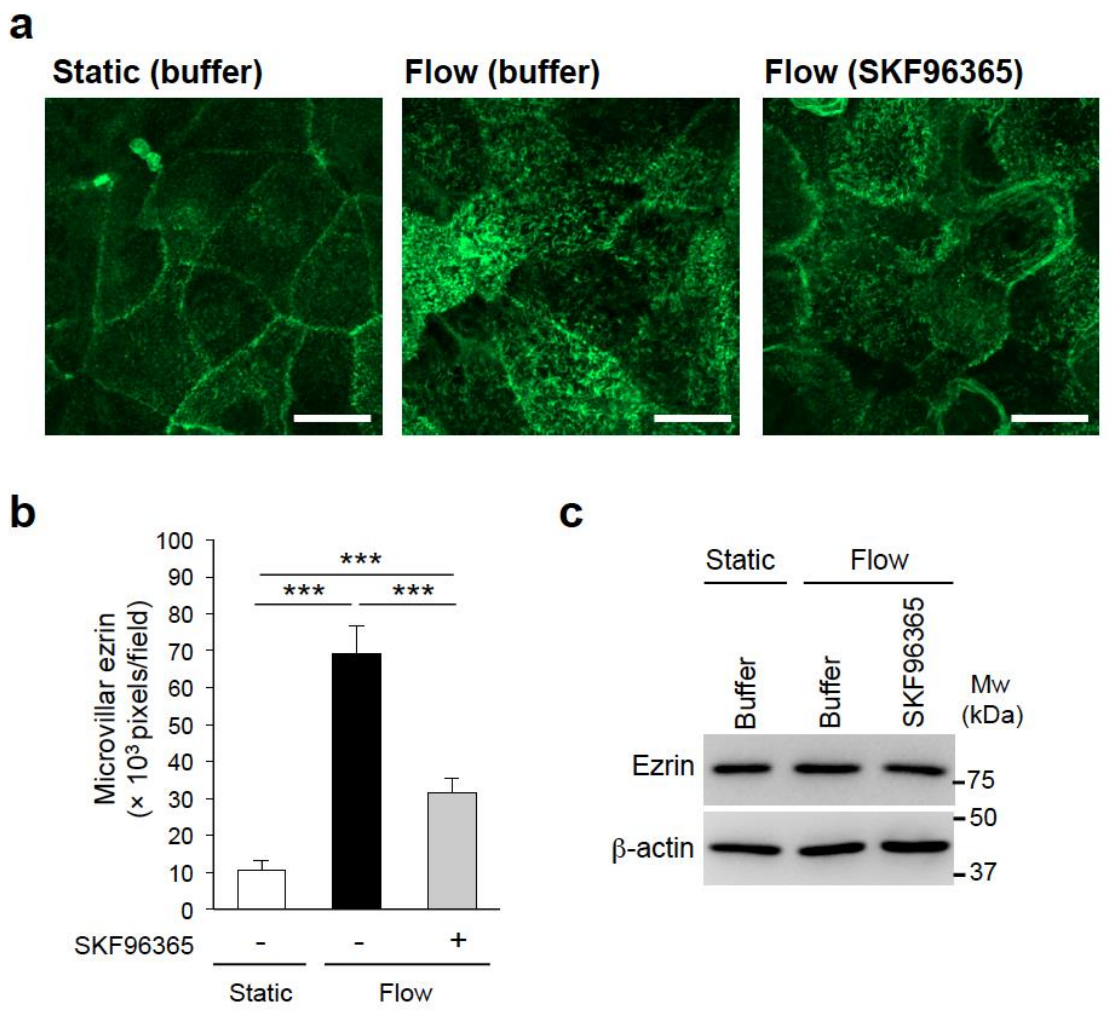

Supplementary Figure 3. Effect of SKF96365 on localization and protein expression level of Ezrin. (**a**) BeWo cells were seeded in the chamber area of the device and cultured for 10 h with medium perfusion (5 µl/min) in the presence or absence (buffer) of SKF96365 (30 µM). The cells were fixed and immunostained with anti-Ezrin antibody. The images are maximum intensity projections of confocal *z*-stacks. Scale bars, 20 µm. (**b**) Quantification of Ezrin re-localization. Microvillar localization of Ezrin was quantified by subtracting Ezrin signals detected at cell-cell contact region form the total signal (five random fields for each condition). The data represent the mean ± s.d.; $***P < 0.001$, analysis of variance. (**c**) The cells were cultured for 12 h in the presence or absence (buffer) of SKF96365 (30 µM) and subjected to immunoblot analysis with anti-Ezrin antibody and anti-β-actin (loading control) antibody.

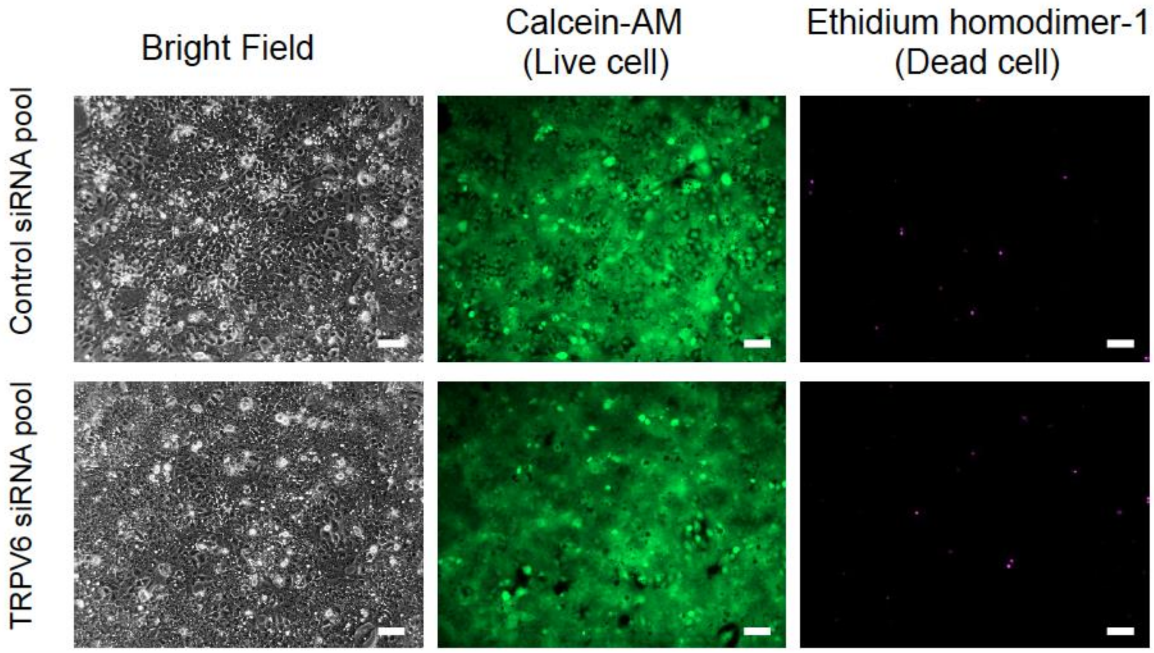

siRNA	The number of dead cell/ field
Control siRNA pool	4.8 ± 1.4
TRPV6 siRNA pool	5.2 ± 2.2
TRPV6 siRNA#1	4.4 ± 2.4
TRPV6 siRNA#2	4.0 ± 1.0
TRPV2 siRNA pool	4.6 ± 1.9

Supplementary Figure 4. Effect of siRNA TRPV6 knockdown on BeWo cell viability. Cells were seeded at a density of 7 × 10^4 cells/well (24-multiwell plate) and cultured overnight prior to siRNA transfection. The transfected cells were cultured for 36 h, and cell viability was analyzed by incubating the cells with Calcein-AM (live cells) and ethidium homodimer-1 (dead cells). Five images were captured under fluorescence microscope (10× objective), and the number of cells positive for ethidium homodimer-1 was counted. The assays were performed in duplicate and repeated twice; similar results were recorded each time. Note that there was no significant increase in the number of dead cells transfected with TRPV6 siRNA compared with the control siRNA. Scale bars, 100 µm.

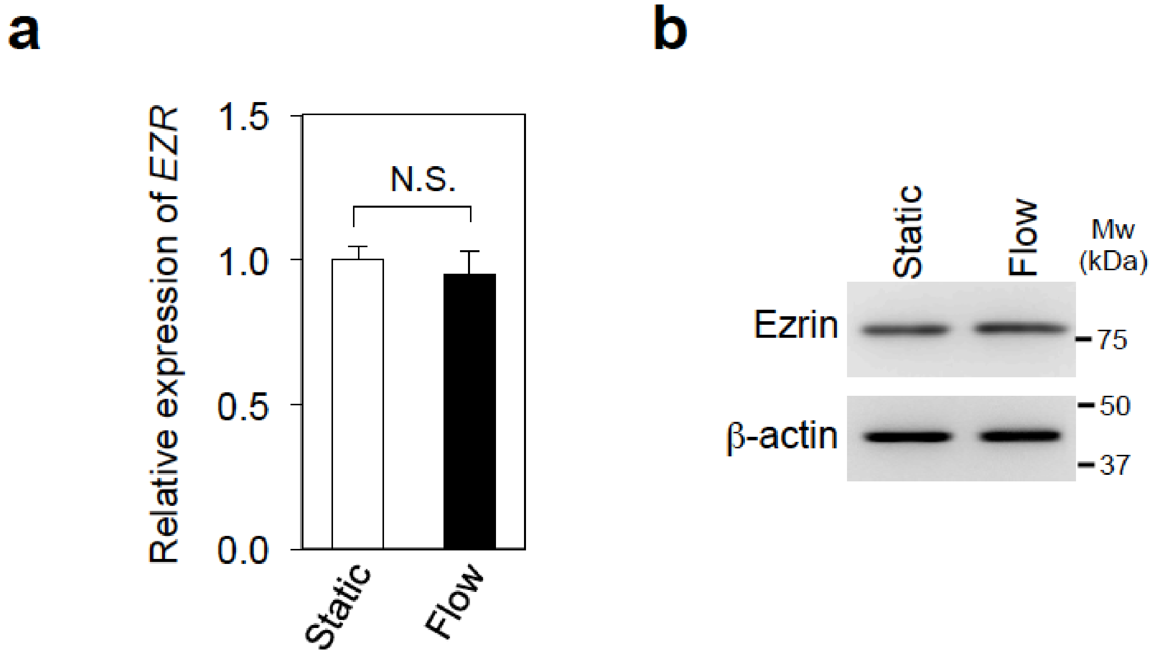

Supplementary Figure 5. Effect of FSS on Ezrin expression. BeWo cells were seeded in the chamber area of the device and culture overnight under static or fluid flow (5 µl/min) condition. The cells were lysed and subjected to real-time PCR (**a**) or immunoblotting (**b**) analysis for Ezrin expression. (**a**) Relative expression of *EZR* normalized to 18S ribosomal RNA level. Data represent the mean ± s.e.m. from three independent experiments. Data significance was assessed by unpaired two-tailed Student's *t*-test; *N.S.*, not significant. (**b**) Cell lysate was analyzed by immunoblotting with anti-Ezrin antibody or anti-β-actin antibody (loading control).

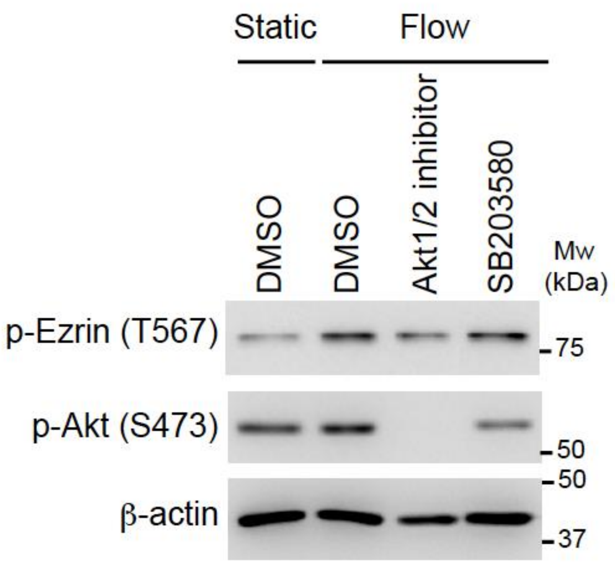

Supplementary Figure 6. Inhibition of Ezrin phosphorylation by the pharmacological inhibitor of Akt1/2. BeWo cells were seeded in the chamber area of the device and cultured overnight under static condition. Culture medium in the device was then replaced with the medium containing a kinase inhibitor of Akt1/2 (Akt inhibitor VIII trifluoroacetate salt hydrate; 10 µM) or SB203580 (an inhibitor of p38 MAP kinase; 10 µM), or DMSO (control), and perfused for 3 h at 5 µl/min. After 3 h of culture, the cells were lysed and subjected to immunoblotting with anti-phospho-Ezrin (Thr567), anti-phospho-Akt (Ser473), or anti-β-actin antibody as a loading control.

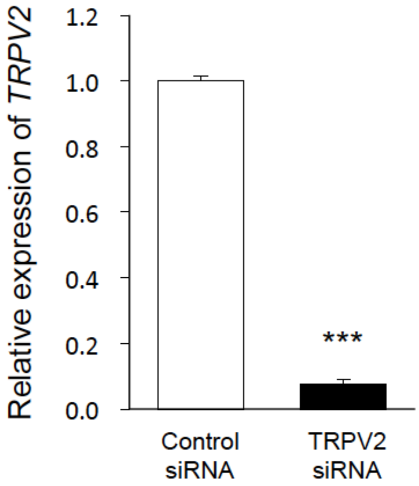

Supplementary Figure 7. TRPV2 knockdown in BeWo cells. Cells were seeded at a density of 7×10^4 cells/well (24-multiwell plate) and cultured overnight prior to siRNA transfection. The transfected cells were cultured for 48 h, and relative expression of *TRPV2* normalized to 18S ribosomal RNA level was analyzed by real-time PCR. Data represent the mean ± s.e.m. from three independent experiments in a triplicate assay. Data significance was assessed by unpaired two-tailed Student's *t*-test; $***P < 0.001$.

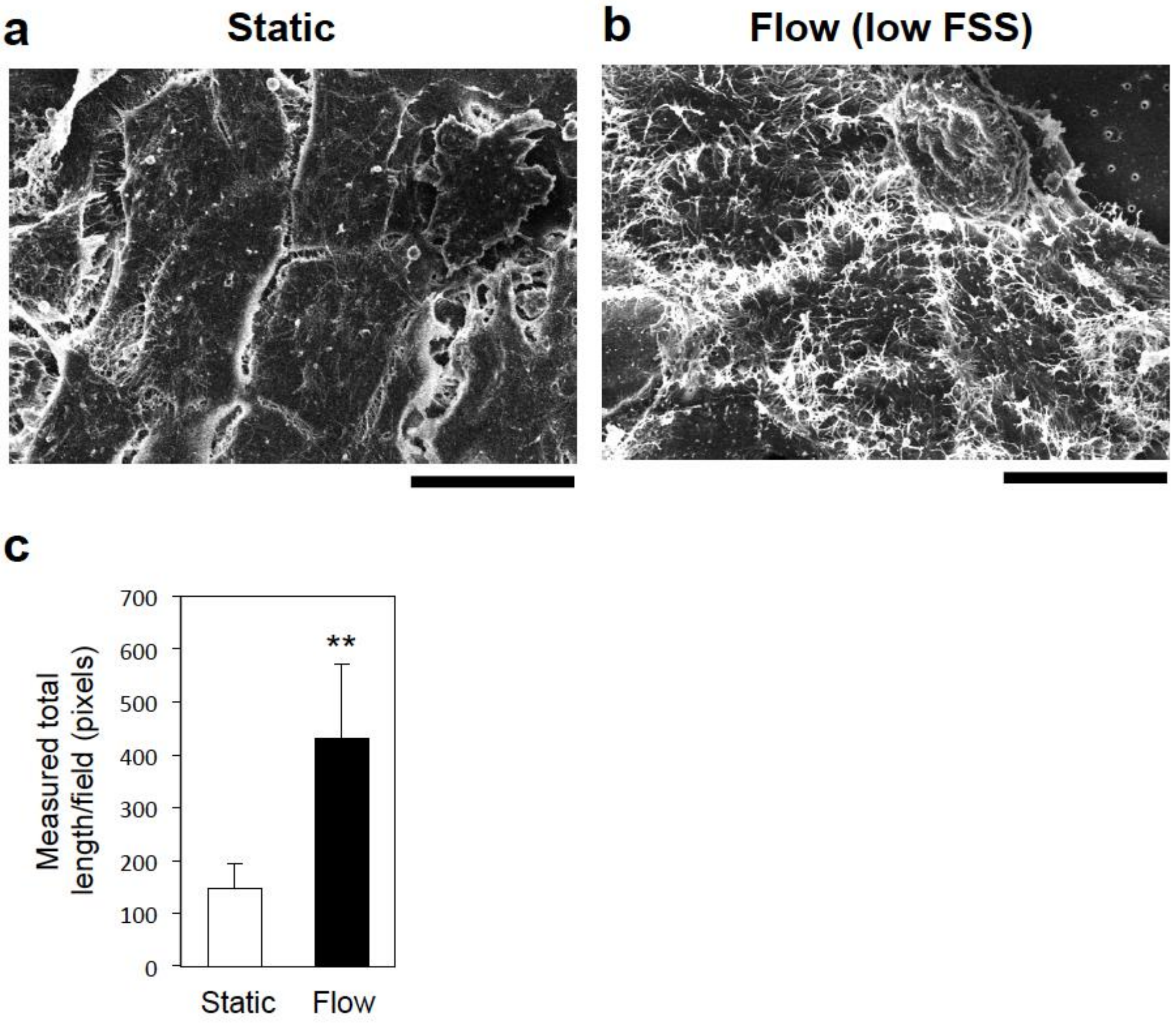

Supplementary Figure 8. FSS induces microvilli formation in BeWo cells cultured on a porous membrane scaffold. Cells were seeded on a porous polyester membrane (0.4-µm pores) scaffold coated with type I collagen (50 µg/ml) and then cultured under static (**a**) or fluid flow (2 µl/min, **b**) conditions. Images were captured at the center area of the chamber. Scale bars, 20 µm. (**c**) Quantification of microvilli. Total length of microvilli/field was measured from the SEM images (2800 μm^2, five fields), as described in METHODS. The data represent the mean ± s.d.; $^{**}P < 0.01$, Student's *t*-test.

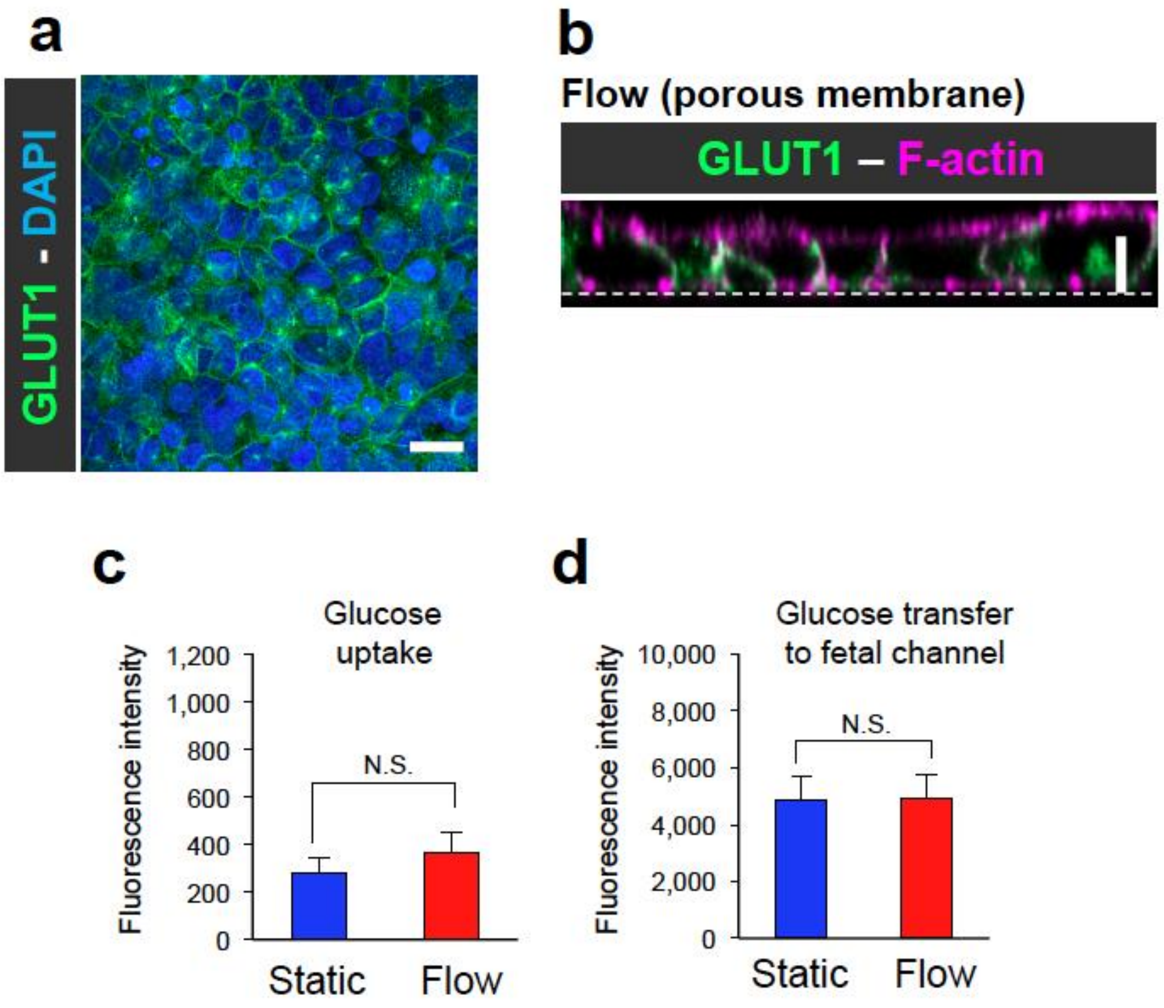

Supplementary Figure 9. GLUT1 localization and glucose transport in BeWo cells cultured on the porous membrane scaffold. Cells were seeded on a porous polyester membrane (0.4 μm pores) scaffold coated with type I collagen (50 μg/ml) and then cultured under static or fluid flow (2 μl/min) conditions. (**a**) Immunofluorescence of GLUT1 (*green*) in BeWo cells cultured under fluid flow (2 μl/min) conditions. Nuclei were counterstained with DAPI (*blue*). Scale bar, 50 μm. (**b**) Representative confocal images of *x-z* optical sections of BeWo cells stained with GLUT1 antibody (*green*) and phalloidin (F-actin, *magenta*). Scale bar, 10 μm. (**c** and **d**) Glucose uptake and transfer to the fetal channel were analyzed by 2-NBDG (2 mM) uptake assays. Data are presented as the mean ± s.e.m. (n = 5 for each condition). Significance was assessed by unpaired two-tailed Student's t-test; *N.S.*, not significant.

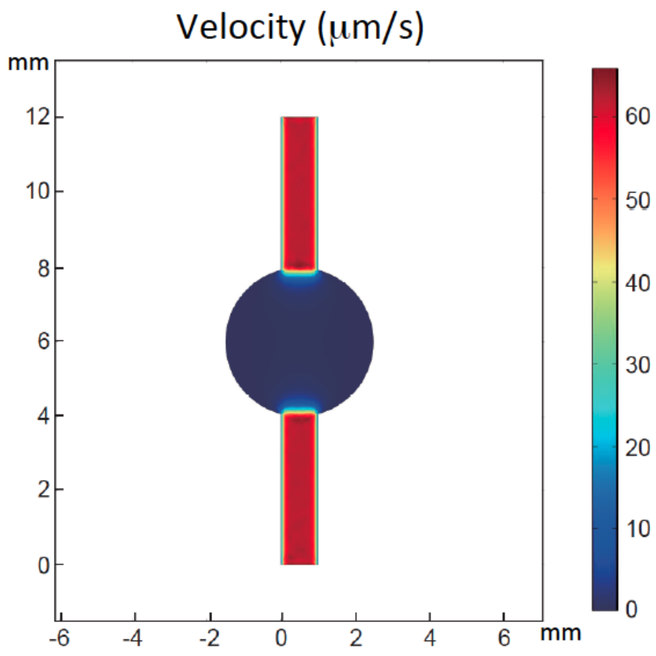

Supplementary Figure 10. Simulation of shear velocity distribution in the maternal chamber. Computational simulation was performed at a flow rate of 5 µl/min. The result of the simulation was a distribution of the shear velocity at the height of 5 µm from the bottom of the maternal channel, assuming that the height of the flowing medium reaches 1.6 mm in the chamber.

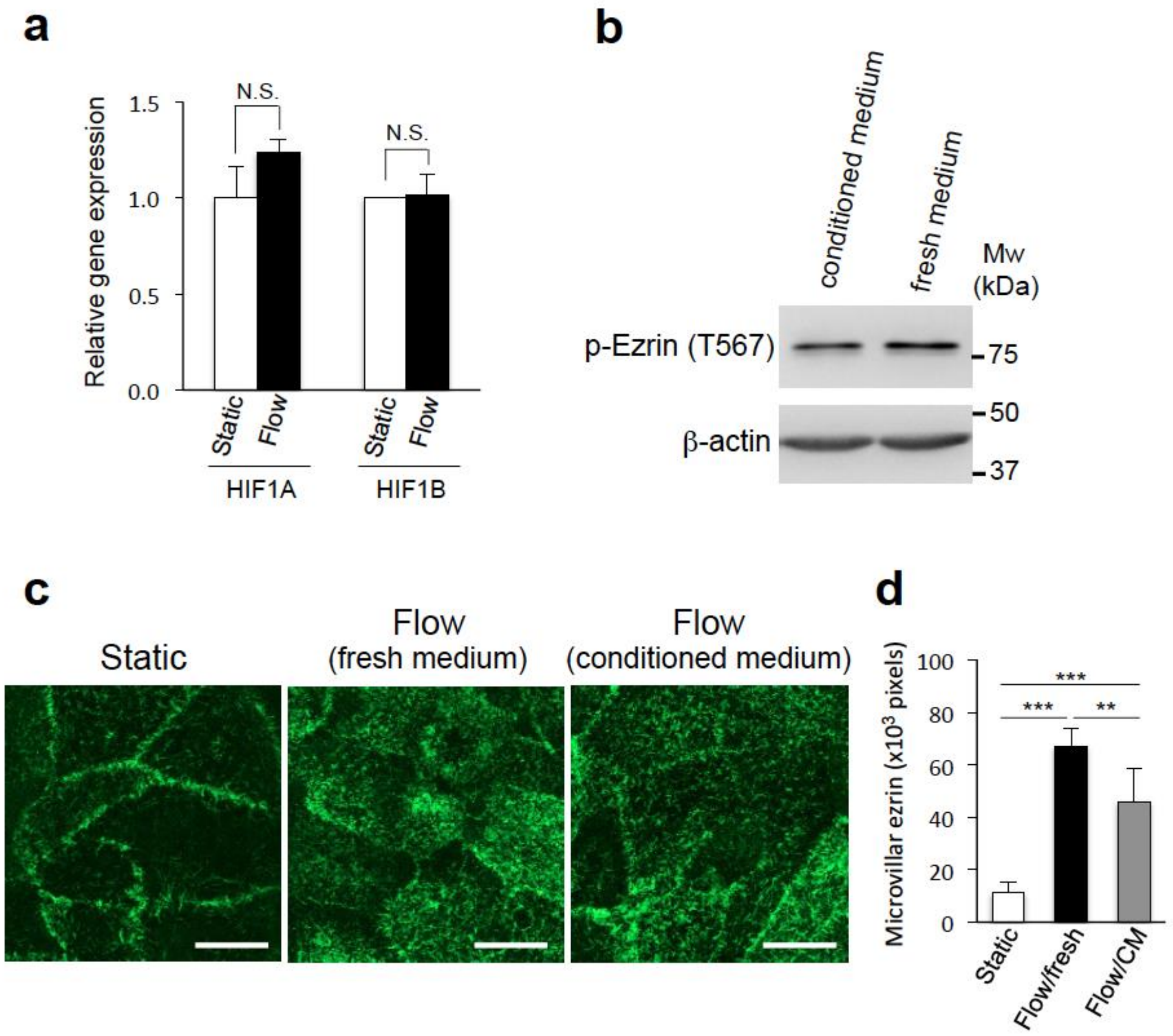

Supplementary Figure 11. Sufficient oxygen and nutrients supply in the cells cultured in microfluidic device. (a) BeWo cells (3.0×10^4 cells) were seeded in the maternal chamber of the device and cultured overnight under static or fluid flow conditions. Relative expression of *HIF1A and HIF1B* normalized to 18S ribosomal RNA level was analyzed by quantitative real-time PCR. Data represent the mean ± s.e.m. from three independent experiments in a triplicate assay. Data significance was assessed by unpaired two-tailed Student's *t*-test; *N.S.*, not significant. (**b**) BeWo cells (3.0×10^4 cells) were seeded in the maternal chamber of the device and cultured overnight under static conditions. The cells in the device were further cultured under static condition with (fresh medium) or without (conditioned medium) medium change. Note that basal levels of Ezrin phosphorylation (Thr567) are not significantly

different. (**c** and **d**) BeWo cells (3.0×10^4 cells) were seeded in the maternal chamber of the device and cultured overnight under static conditions. Fresh culture medium or conditioned medium (10 ml of culture medium incubated overnight with confluent BeWo cells in 100 mm culture dish) was then perfused (5 μl/min) for 1 h prior to the fixation of cells. Microvillar localization of Ezrin was analyzed by immunostaining (**c**) and quantified using ImageJ from five random fields (**d**). The data represent the mean ± s.d.; $^{**}P < 0.01$, $^{***}P < 0.001$, analysis of variance. Scale bars, 20 µm.

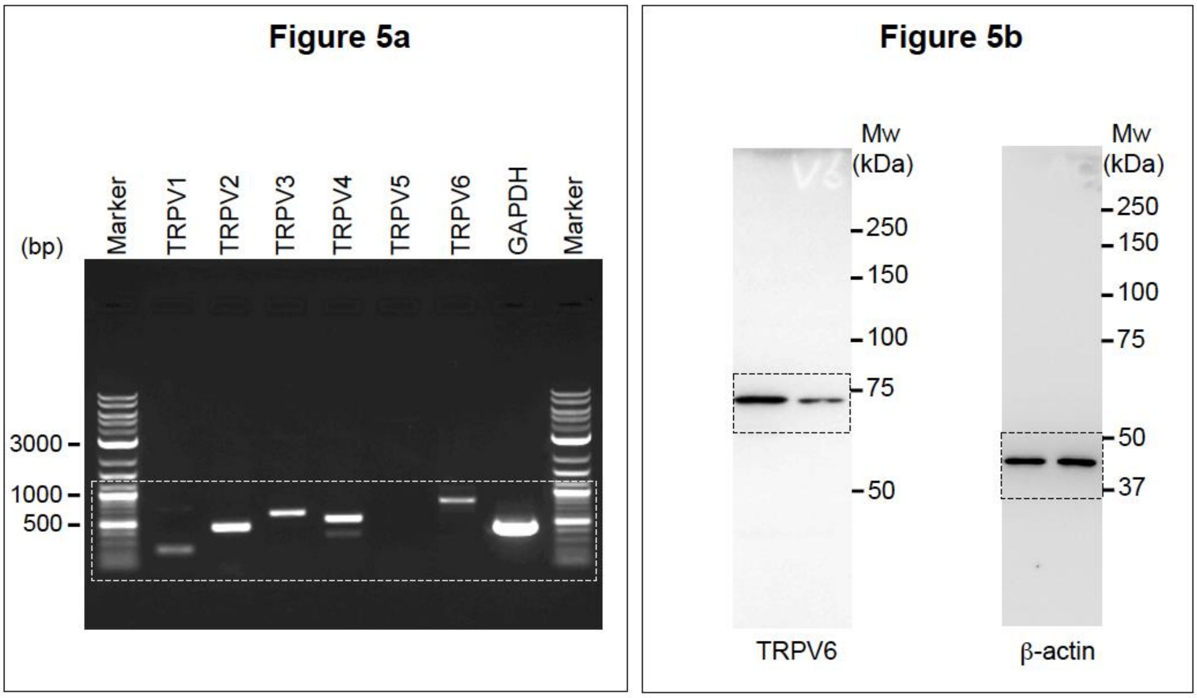

Supplementary Figure 12. Full images of DNA gel electrophoresis and immunoblots in the main manuscript (Figure 5).

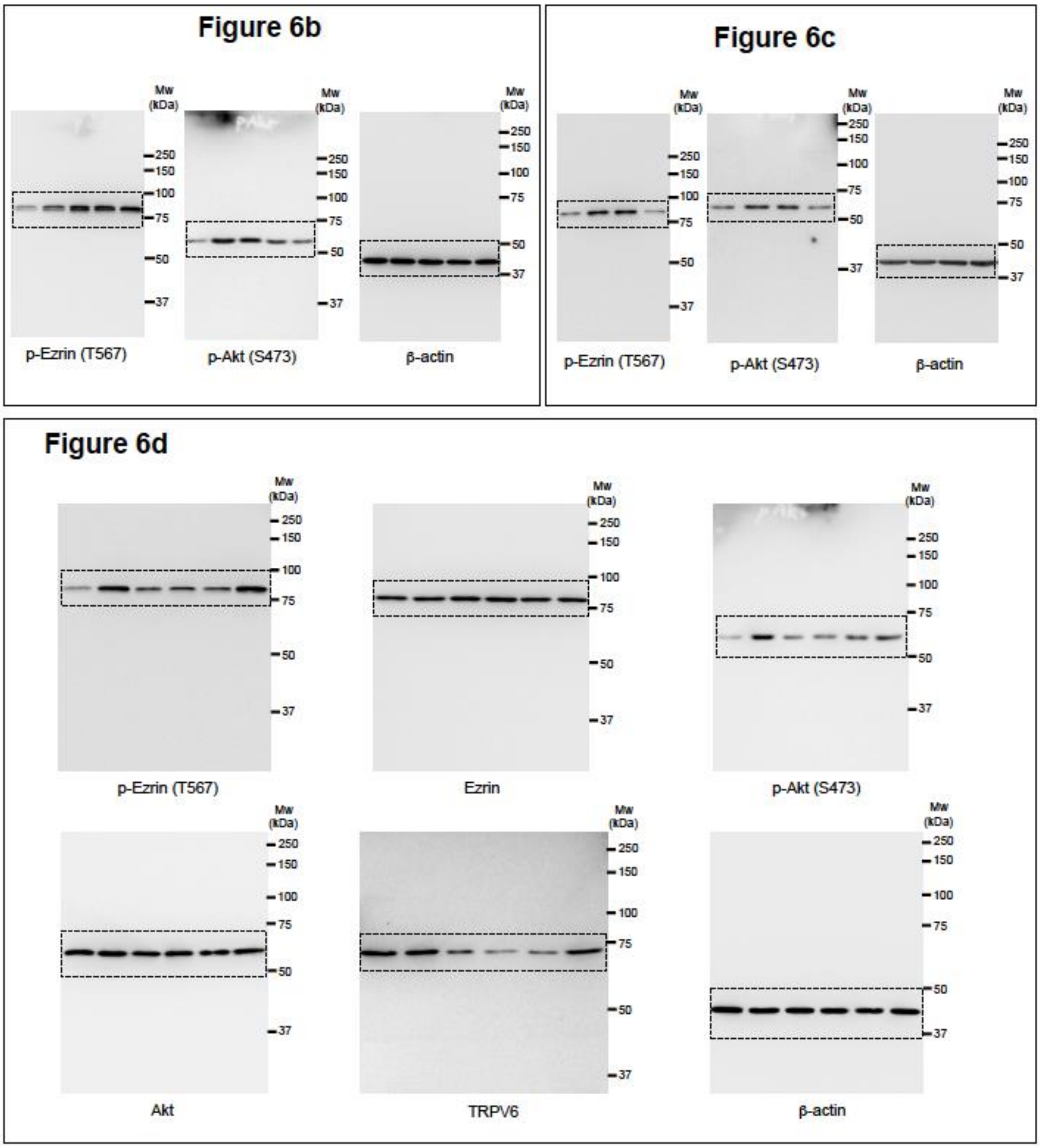

Supplementary Figure 13. Full images of immunoblots in the main manuscript (Figure 6).

SUPPLEMENTARY METHODS

Materials and antibodies

Anti-dynein, axonemal, light chain 4 (DNAL4) antibody (1:200, HPA003647) was purchased from Sigma-Aldrich (St Louis, MO). SKF96365 (a general inhibitor of receptor operated calcium entry), SB203580 (p38 MAP kinase inhibitor), and Akt1/2 inhibitor (Akt inhibitor VIII) were from Wako Pure Chemical (Osaka, Japan). Capsaicin was purchased from Sigma-Aldrich. LIVE & DEAD Assay Kit for the evaluation of cell viability was derived from Life Technologies (Carlsbad, CA).

Quantitative real-time PCR analysis

Quantitative real-time PCR analyses were performed using TaqMan Fast Advanced Master Mix and TaqMan Gene Expression assays (*EZR*, Hs00931653_m1 and *RN18S1*, Hs03928985_g1 as internal controls) or SYBR Premix Ex Taq II (TaKaRa Bio, Japan) on a StepOne Real-Time PCR system (Applied Biosystems/Life Technologies, Carlsbad, CA). Reactions were run in triplicate and analyzed by the comparative C_T method. Specific primers used for *TRPV2*, *HIF1A*, and *HIF1B* expression analysis were as follows: human *TRPV2* forward primer: 5′-TCAGGTTGGAGACATTAGATGGA-3′; human *TRPV2* reverse primer: 5′-TCGGTAGTTGAGGTTGACTCTT-3′; human *HIF1A* forward primer: 5′-TTTTTCAAG CAGTAGGAATTGGA-3′; human *HIF1A* reverse primer: 5′-GTGATGTAGTAGCTGCAT GATCG-3′; human *HIF1B* forward primer: 5′-CTGCCAACCCCGAAATGACAT-3′; human *HIF1B* reverse primer: 5′-CGCCGCTTAATAGCCCTCTG-3′; human *RPS18* forward primer: 5′-GCGGCGGAAAATAGCCTTTG-3′; and human *RPS18* reverse primer: 5′-GATCACACGTTCCACCTCATC-3′.
